# Real-world analysis of leuprorelin acetate microspheres-based neoadjuvant therapy for patients with high-risk prostate cancer

**DOI:** 10.3389/fonc.2025.1520370

**Published:** 2025-03-19

**Authors:** Changde Fu, Jun Xin, Jinjin Lai, Xu Zeng, Yongnan Wang, Wei Zhang

**Affiliations:** Department of Urology, Quanzhou First Hospital Affiliated to Fujian Medical University, Quanzhou, China

**Keywords:** leuprorelin, high-risk prostatic cancer, neoadjuvant therapy, prostate-specific antigen, testosterone

## Abstract

**Objective:**

Boennuokang^®^ leuprorelin acetate microspheres show a certain efficacy in patients with prostate cancer, but its utilization as neoadjuvant therapy in patients with high-risk prostate cancer remains unclear. Hence, this real-world study investigated the efficacy and safety of Boennuokang^®^ leuprorelin acetate microspheres-based treatment as neoadjuvant therapy in patients with high-risk prostate cancer.

**Methods:**

This retrospective study included 53 patients with high-risk prostate cancer who received Boennuokang^®^ leuprorelin acetate microspheres as neoadjuvant therapy and laparoscopic radical prostatectomy.

**Results:**

The median prostate-specific antigen (PSA) was 34.1 ng/mL before neoadjuvant therapy and reduced to 0.8 ng/mL after neoadjuvant therapy (*P*<0.001). Testosterone showed a decreased tendency after neoadjuvant therapy, but without statistical significance (*P*=0.185). After surgery, 36 (67.9%) patients had negative surgical margin. The median (interquartile range) prostate volume reduced from 40.5 (33.4-55.2) mL before neoadjuvant therapy to 30.2 (25.2-40.2) mL after neoadjuvant therapy (*P*<0.001). Meanwhile, alkaline phosphatase before neoadjuvant therapy, at one month (M1), 3 months (M3), 6 months (M6), and 12 months (M12) after surgery tended to be increased (*P*=0.029), but this increment lacks clinical significance, while the glomerular filtration rate (*P*=0.441) and albumin (*P*=0.548) did not vary among different time points. Erectile dysfunction and loss of libido was the most common adverse event, with incidences of 84.9% during neoadjuvant therapy, 79.2% at M1, 71.7% at M3, 67.9% at M6, and 56.6% at M12.

**Conclusion:**

Boennuokang^®^ leuprorelin acetate microspheres-based treatment as neoadjuvant therapy decreases PSA, testosterone, and prostate volume, with acceptable positive surgical margin rate in patients with high-risk prostate cancer and its safety profiles should be validated.

## 1 Introduction

Prostatic cancer is the second most common cancer in men, with an estimation of 1.5 million new cases globally in 2022 (1). Although most patients with prostate cancer are diagnosed with low-risk disease, approximately 15% of the patients are identified with high-risk prostatic cancer and face poor prognosis, with a 5-year biochemical recurrence (defined as prostate-specific antigen (PSA) ≥0.2 ng/mL)-free rate of 41.6%-62.8% (2–6). Radical prostatectomy is a standard treatment for prostatic cancer; nevertheless, some high-risk prostatic patients are ineligible for the surgery due to significant tumor burden (3). Therefore, neoadjuvant therapy is warranted to improve outcomes for patients with high-risk prostate cancer.

Leuprorelin, a luteinizing hormone-releasing hormone (LHRH) agonist, inhibits the production of androgen and is recommended as androgen deprivation therapy in combination with antiandrogen drugs, such as abiraterone, bicalutamide, and flutamide (7, 8). The efficacy of leuprorelin-based treatment as neoadjuvant therapy in patients with high-risk prostate cancer has been demonstrated by previous clinical trials (9–12). For instance, two previous randomized, controlled trials indicated that leuprorelin-based treatment as neoadjuvant therapy reduced PSA and testosterone levels in patients with high-risk prostate cancer (9, 10). According to another study, the 3-year biochemical progression-free survival rate was 81% and 72% after 12- and 6-month leuprolide-based neoadjuvant therapy in patients with high-risk prostate cancer (12).

Boennuokang^®^ leuprorelin acetate microspheres is the first generic product in China developed by Beijing Biote Pharmaceutical Co., Ltd., which shows consistent efficacy with branded leuprorelin acetate microspheres in patients with prostate cancer (13). According to previous studies, Boennuokang^®^ leuprorelin acetate microspheres indicated a certain effect on reducing the PSA and testosterone level in patients with prostate cancer (13, 14). However, the real-world evidence on the efficacy of Boennuokang^®^ leuprorelin acetate microspheres-based treatment as neoadjuvant therapy in patients with high-risk prostate cancer remains unclear.

Hence, this real-world study aimed to assess the efficacy and safety of Boennuokang^®^ leuprorelin acetate microspheres-based treatment as neoadjuvant therapy in patients with high-risk prostate cancer.

## 2 Methods

### 2.1 Patients

A total of 53 patients with high-risk prostate cancer who received Boennuokang^®^ leuprorelin acetate microspheres-based treatment as neoadjuvant therapy and laparoscopic radical prostatectomy from November 2013 to November 2023 were screened for this retrospective study. The study design was shown in **Supplementary Figure 1**. The inclusion criteria contained: a) diagnosed as prostatic cancer; b) confirmed as high-risk prostatic cancer, which was defined as cT2c-4, PSA >20 ng/mL, or Gleason score ≥8 (15); c) received Boennuokang^®^ leuprorelin acetate microspheres (Beijing Biote Pharmaceutical Co., Ltd.) as neoadjuvant therapy and laparoscopic radical prostatectomy; d) had available clinical characteristics information for analysis. The exclusion criteria contained: a) had prior radiotherapy or chemotherapy; b) had other malignant tumors; c) had underlying diseases that were seriously unable to tolerate surgery or had a significant impact on the surgical effect; d) had a history of pelvic and bladder neck surgery. The Ethics Committee permitted the study. Each patient or family member offered informed consent.

### 2.2 Collection of baseline characteristics

The baseline characteristics were collected from the electronic medical data system, which contained age, body mass index, smoke history, hypertension, diabetes, clinical tumor (T) stage, Gleason score, and prostate volume.

### 2.3 Collection of neoadjuvant and surgical parameters

The neoadjuvant and surgical parameters were backtracked, which contained neoadjuvant therapy duration, neoadjuvant therapy regimen, prostate volume after neoadjuvant therapy, pathological T stage, operation time, intraoperative blood loss, positive surgical margin, urine control recovery time, and hospital stays.

For neoadjuvant therapy, Boennuokang^®^ leuprorelin acetate microspheres was given subcutaneously at 3.75 mg every 28 days, and antiandrogen drugs such as abiraterone, bicalutamide, and flutamide were also given as needed. Abiraterone acetate was given orally at 1000 mg once daily combined with prednisone 5mg orally, twice daily. Bicalutamide was given orally at 50 mg once daily. Flutamide was given orally at 250 mg once every 8 hours.

The prostate volume was measured using ultrasound and the calculation method was as follows: prostate volume = (length × width × height) × 0.52.

Urine control recovery was defined as smooth urination after catheter removal and using ≤1 pad every day. Urinary control recovery time was defined as the time during which patients could urinate on their own with the use of ≤1 pad every day after surgery. The evaluation time points were set at 1 week after catheter removal, with patients instructed to record their urination status. Subsequent evaluations were conducted every 1-2 weeks or according to the patient’s specific urination condition, based on clinical judgment.

### 2.4 Collection of follow-up data

PSA and testosterone before neoadjuvant therapy, after neoadjuvant therapy (which was defined as before surgery), at one month after surgery (M1), at 3 months after surgery (M3), at 6 months after surgery (M6), and at 12 months after surgery (M12) were collected. Biochemical recurrence rate was defined as two episodes of PSA≥0.2 ng/mL after surgery, which was assessed at M3, M6, and M12 (16). Glomerular filtration rate (GFR), alkaline phosphatase, and albumin before neoadjuvant therapy, at M1, at M3, at M6, and at M12 were also backtracked. Besides, the adverse events during neoadjuvant therapy, at M1, at M3, at M6, and at M12 were also collected for safety assessment.

### 2.5 Statistics

The SPSS v.26.0 from IBM, USA was used for statistics. Data were shown using mean ± standard deviation, No. (%), minimum, maximum, or median (25^th^-75^th^) as appropriate. Comparisons of PSA, testosterone, GFR, alkaline phosphatase, and albumin at different time were analyzed using the Friedman test. The *post-hoc* comparisons of PSA, testosterone, GFR, alkaline phosphatase, and albumin between different time and before neoadjuvant therapy were analyzed using Wilcoxon signed-rank test. Subgroup analyses was performed using Fisher’s exact test or Friedman test. A *P* value <0.05 (two-sided) indicated significance.

## 3 Results

### 3.1 Clinical characteristics

The mean age of patients was 67.3 ± 6.2 years. All the patients had prostatic adenocarcinoma. In total, 7 (13.2%), 28 (52.8%), 15 (28.3%), and 3 (5.7%) patients were at clinical T1, T2, T3, and T4 stage, respectively. Regarding Gleason grade, 2 (3.8%), 3 (5.7%), 16 (30.2%), 9 (17.0%), and 23 (43.4%) patients were at Gleason grade 1, 2, 3, 4, and 5, respectively. The detailed baseline characteristics are listed in **Table 1**.

**Table 1:** Baseline characteristics.

Items	Patients (N = 53)
Age (years)	67.3 ± 6.2
BMI (kg/m^2^)	23.4 ± 3.2
Smoke history	6 (11.3)
Hypertension	13 (24.5)
Diabetes	6 (11.3)
Histological Type	
adenocarcinoma	53 (100)
Clinical T stage	
T1	7 (13.2)
T2	28 (52.8)
T3	15 (28.3)
T4	3 (5.7)
Gleason grade	
1 (3 + 3)	2 (3.8)
2 (3 + 4)	3 (5.7)
2 (4 + 3)	16 (30.2)
4 (4 + 4, 3 + 5, 5 + 3)	9 (17.0)
5 (4 + 5, 5 + 4, 5 + 5)	23 (43.4)

Data were shown using mean ± standard deviation, No. (%), or median (25^th^-75^th^). BMI, body mass index; T, tumor.

### 3.2 PSA and testosterone at different time points

**Figure 1:**
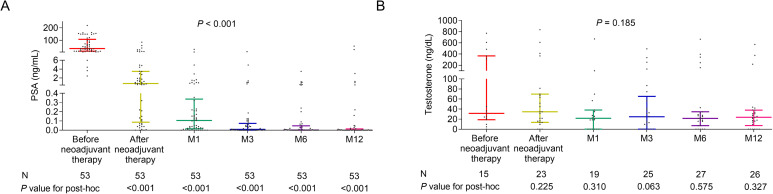
Levels and trends of PSA and testosterone over time in patients with high-risk prostate cancer who received Boennuokang^®^ leuprorelin acetate microspheres-based treatment as neoadjuvant therapy. PSA **(A)** and testosterone **(B)** levels before neoadjuvant therapy, after neoadjuvant therapy, at M1, at M3, at M6, and at M12.

PSA before neoadjuvant therapy (N=53), after neoadjuvant therapy (N=53), at M1 (N=53), at M3 (N=53), at M6 (N=53), and at M12 (N=53) varied and showed a reduction over time (*P*<0.001). PSA after neoadjuvant therapy, at M1, at M3, at M6, and at M12 was lower than its level before neoadjuvant therapy (all *P*<0.001) (**Figure 1**). The details of PSA level at 12 months post-surgery were listed in **Table 2**. The 3-month, 6-month, and 12-month biochemical recurrence rate was 9.4%, 5.7%, and 7.5%, respectively. The details of baseline Gleason grade in patients with biochemical recurrence was listed in **Supplementary Table 1**.

**Table 2:** PSA levels.

Items	Before neoadjuvant therapy	After neoadjuvant therapy	M1	M3	M6	M12
Minimum, ng/mL	2.5	0.0	0.0	0.0	0.0	0.0
25th, ng/mL	12.7	0.1	0.0	0.0	0.0	0.0
Median, ng/mL	34.1	0.8	0.1	0.0	0.0	0.0
75th, ng/mL	108.0	3.5	0.3	0.1	0.0	0.0
Maximum, ng/mL	218.4	84.4	27.5	9.1	3.5	53.7
Biochemical recurrence rate, n (%)	NA	NA	NA	5 (9.4)	3 (5.7)	4 (7.5)

Data were shown using No. (%) or median (25^th^-75^th^). PSA, prostate-specific antigen; M1, 1 month after surgery; M3, 3 months after surgery; M6, 6 months after surgery; M12, 12 months after surgery; NA, Not Applicable.

Testosterone before neoadjuvant therapy (N=15), after neoadjuvant therapy (N=23), at M1 (N=19), at M3 (N=25), at M6 (N=27), and at M12 (N=26) was also evaluated and its level tended to decrease after neoadjuvant therapy, but did not vary over time (*P*=0.185) (**Figure 1**).

### 3.3 Neoadjuvant therapy and surgical outcomes

The median (25^th^-75^th^) neoadjuvant therapy duration was 3.0 (3.0-4.0) months. Forty-seven (88.7%) patients received Boennuokang^®^ leuprorelin acetate microspheres plus bicalutamide, 5 (9.4%) patients received Boennuokang^®^ leuprorelin acetate microspheres plus abiraterone, and one (1.9%) patient was administrated Boennuokang^®^ leuprorelin acetate microspheres plus flutamide. After neoadjuvant therapy, 10 (18.9%) patients in this study achieve downstaging at surgery. The detailed information of patients with downstaging at surgery was displayed in **Supplementary Table 2**. In addition, the median (25^th^-75^th^) operation time was 2.4 (2.0-3.4) hours and intraoperative blood loss was 50.0 (50.0-100.0) mL. The median (25^th^-75^th^) urine control recovery time and hospital stay were 3.0 (2.5-5.0) days and 14.0 (8.5-19.0) days, respectively (**Table 3**).

**Table 3:** Neoadjuvant and surgical parameters.

Items	Patients (N = 53)
Neoadjuvant therapy duration (months)	3.0 (3.0-4.0)
Neoadjuvant therapy regimen	
Boennuokang^®^ leuprelin acetate microspheres plus bicalutamide	47 (88.7)
Boennuokang^®^ leuprelin acetate microspheres plus abiraterone	5 (9.4)
Boennuokang^®^ leuprelin acetate microspheres plus flutamide	1 (1.9)
Downstaging at surgery	10 (18.9)
Operation time (hours)	2.4 (2.0-3.4)
Intraoperative blood loss (mL)	50.0 (50.0-100.0)
Urine control recovery time (days)	3.0 (2.5-5.0)
Hospital stays (days)	14.0 (8.5-19.0)

Data were shown using No. (%) or median (25^th^-75^th^).

### 3.4 Positive surgical margin and change of prostate volume

**Figure 2:**
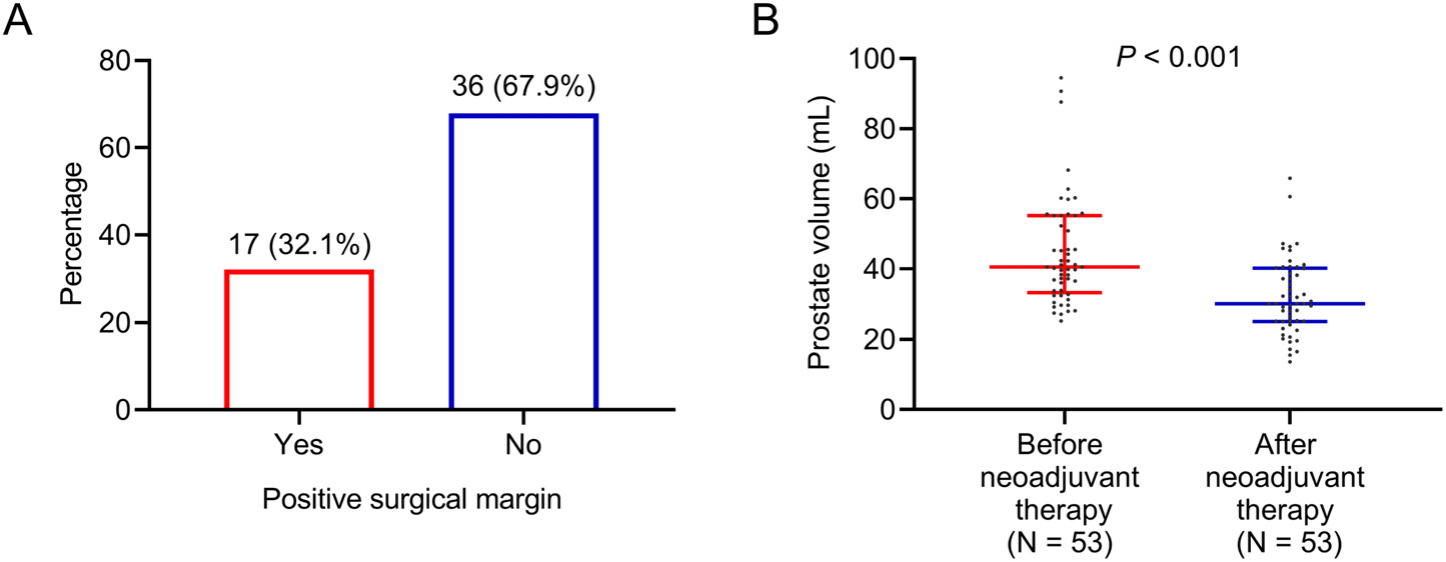
Positive surgical margin and prostate volume in patients with high-risk prostate cancer who received Boennuokang^®^ leuprorelin acetate microspheres-based treatment as neoadjuvant therapy. Positive surgical margin **(A)** and prostate volume before and after neoadjuvant therapy **(B)**.

After surgery, 36 (67.9%) patients had negative surgical margin, while 17 (32.1%) patients still had positive surgical margin (**Figure 2**). Additionally, prostate volume was decreased after neoadjuvant therapy (*P*<0.001). In detail, the median (25^th^-75^th^) prostate volume before (N=53) and after (N=53) neoadjuvant therapy were 40.5 (33.4-55.2) mL and 30.2 (25.2-40.2) mL, respectively (**Figure 2**).

### 3.5 Subgroup analyses based on neoadjuvant therapy regimen

In subgroup of Boennuokang^®^ leuprelin acetate microspheres plus bicalutamide, PSA showed a reduction over time after neoadjuvant therapy (*P* **<**0.001). Similarly, in subgroup of Boennuokang^®^ leuprelin acetate microspheres plus abiraterone, PSA was decreased over time after neoadjuvant therapy (*P*=0.012). The comparison of PSA was not applicable in subgroup of Boennuokang^®^ leuprelin acetate microspheres plus flutamide. Regarding testosterone level in the subgroup of Boennuokang^®^ leuprelin acetate microspheres plus bicalutamide, it did not vary before neoadjuvant therapy, after neoadjuvant therapy, at M1, M3, M6, and M12 (*P*=0.201). The comparison of testosterone was not applicable in subgroup of Boennuokang^®^ leuprelin acetate microspheres plus abiraterone or Boennuokang^®^ leuprelin acetate microspheres plus flutamide. Biochemical recurrence rate was 10.6% at M3, 6.4% at M6, and 8.5% at M12 in the subgroup of Boennuokang^®^ leuprelin acetate microspheres plus bicalutamide. In subgroup of Boennuokang^®^ leuprelin acetate microspheres plus bicalutamide and Boennuokang^®^ leuprelin acetate microspheres plus flutamide, no patient had biochemical recurrence (**Table 4**).

**Table 4:** Subgroup analysis based on neoadjuvant therapy regimen.

Items	Before neoadjuvant therapy	After neoadjuvant therapy	M1	M3	M6	M12	*P* value for trend
PSA (ng/mL), median (minimum, maximum)							
Subgroup of Boennuokang^®^ leuprelin acetate microspheres plus bicalutamide	31.7 (2.5, 160.0)	0.7 (0.0, 84.4)	0.1 (0.0, 27.5)	0.0 (0.0, 4.9)	0.0 (0.0, 1.2)	0.0 (0.0, 53.7)	<0.001
Subgroup of Boennuokang^®^ leuprelin acetate microspheres plus abiraterone	40.0 (12.0, 218.4)	1.5 (0.0, 23.6)	0.2 (0.0, 0.8)	0.0 (0.0, 9.1)	0.1 (0.0, 3.5)	0.0 (0.0, 1.0)	0.012
Subgroup of Boennuokang^®^ leuprelin acetate microspheres plus flutamide*	151.0	10.1	0.0	0.0	0.0	0.0	NA
Testosterone (ng/dL), median (minimum, maximum)							
Subgroup of Boennuokang^®^ leuprelin acetate microspheres plus bicalutamide	31.5 (0.0, 311.1)	31.5 (16.8, 63.3)	34.9 (0.1, 38.2)	16.2 (0.1, 31.5)	15.9 (0.1, 17.9)	0.1 (0.0, 30.7)	0.201
Subgroup of Boennuokang^®^ leuprelin acetate microspheres plus abiraterone	14.2 (9.6, 18.8)	4.8 (0.1, 9.6)	18.8 (0.1, 56.9)	0.1 (0.1, 9.6)	156.7 (0.1, 344.7)	9.6 (0.1, 42.2)	NA
Subgroup of Boennuokang^®^ leuprelin acetate microspheres plus flutamide*	NA	NA	0.0	0.0	NA	NA	NA
Biochemical recurrence rate, n (%)							
Subgroup of Boennuokang^®^ leuprelin acetate microspheres plus bicalutamide	NA	NA	NA	5 (10.6)	3 (6.4)	4 (8.5)	NA
Subgroup of Boennuokang^®^ leuprelin acetate microspheres plus abiraterone	NA	NA	NA	0 (0.0)	0 (0.0)	0 (0.0)	NA
Subgroup of Boennuokang^®^ leuprelin acetate microspheres plus flutamide	NA	NA	NA	0 (0.0)	0 (0.0)	0 (0.0)	NA

*, only one patient in the subgroup of Boennuokang^®^ leuprelin acetate microspheres plus flutamide. The comparison of PSA was not applicable in subgroup of Boennuokang^®^ leuprelin acetate microspheres plus flutamide. The comparison of testosterone was not applicable in subgroup of Boennuokang^®^ leuprelin acetate microspheres plus abiraterone or Boennuokang^®^ leuprelin acetate microspheres plus flutamide. Data were shown using No. (%) or median (minimum, maximum). PSA, prostate-specific antigen; M1, 1 month after surgery; M3, 3 months after surgery; M6, 6 months after surgery; M12, 12 months after surgery; NA, Not Applicable.

### 3.6 Subgroup analyses based on neoadjuvant therapy regimen and Gleason grade

In subgroup of Boennuokang^®^ leuprelin acetate microspheres plus bicalutamide, 2 (4.3%), 3 (6.4%), 14 (29.8%), 7 (14.9%), and 21 (44.7%) patients were assessed with Gleason grade 1 (3 + 3), 2 (3 + 4), 2 (4 + 3), 4 (4 + 4, 3 + 5, 5 + 3), and 5 (4 + 5, 5 + 4, 5 + 5), respectively. In subgroup of Boennuokang^®^ leuprelin acetate microspheres plus abiraterone, 2 (40.0%), 2 (40.0%), and one (20.0%) patient had Gleason grade 2 (4 + 3), 4 (4 + 4, 3 + 5, 5 + 3), and 5 (4 + 5, 5 + 4, 5 + 5), respectively. Subgroup of Boennuokang^®^ leuprelin acetate microspheres plus flutamide only included one patient with Gleason grade 5 (4 + 5, 5 + 4, 5 + 5). Neoadjuvant therapy regimen was not associated with Gleason grades (*P*=0.715) (**Supplementary Table 3**).

In subgroup of Boennuokang^®^ leuprelin acetate microspheres plus bicalutamide, PSA was decreased over time in patients with Gleason grade 2 (4 + 3), 4 (4 + 4, 3 + 5, 5 + 3), and 5 (4 + 5, 5 + 4, 5 + 5), respectively (all *P*<0.001). Regarding testosterone level, the data was missing in subgroup of Boennuokang^®^ leuprelin acetate microspheres plus abiraterone. In subgroup of Boennuokang^®^ leuprelin acetate microspheres plus abiraterone, the changes in PSA did not achieve statistical significance in patients with Gleason grade 2 (4 + 3) and 4 (4 + 4, 3 + 5, 5 + 3) (both *P*>0.05) (**Table 5**).

**Table 5:** Subgroup analysis based on neoadjuvant therapy regimen and Gleason grade.

Items	PSA (ng/mL), median (minimum, maximum)						*P* value for trend
	Before neoadjuvant therapy	After neoadjuvant therapy	M1	M3	M6	M12	
Subgroup of Boennuokang^®^ leuprelin acetate microspheres plus bicalutamide (n = 47)							
Gleason grade of 1 (3 + 3)	18.6 (7.9, 29.2)	0.9 (0.1, 1.7)	0.0 (0.0, 0.0)	0.0 (0.0, 0.0)	0.0 (0.0, 0.0)	0.0 (0.0, 0.0)	0.096
Gleason grade of 2 (3 + 4)	12.9 (9.8, 30.3)	0.5 (0.0, 1.0)	0.1 (0.0, 0.1)	0.0 (0.0, 0.1)	0.0 (0.0, 0.1)	0.0 (0.0, 0.0)	0.062
Gleason grade of 2 (4 + 3)	28.3 (2.5, 160.0)	2.2 (0.0, 23.1)	0.2 (0.0, 6.1)	0.0 (0.0, 1.0)	0.0 (0.0, 1.0)	0.0 (0.0, 53.7)	<0.001
Gleason grade of 4 (4 + 4, 3 + 5, 5 + 3)	11.2 (3.7, 93.9)	0.1 (0.0, 8.4)	0.0 (0.0, 1.0)	0.0 (0.0, 1.3)	0.0 (0.0, 0.2)	0.0 (0.0, 0.5)	<0.001
Gleason grade of 5 (4 + 5, 5 + 4, 5 + 5)	67.0 (8.9, 155.0)	0.7 (0.0, 84.4)	0.1 (0.0, 27.5)	0.0 (0.0, 4.9)	0.0 (0.0, 1.2)	0.0 (0.0, 26.0)	<0.001
Subgroup of Boennuokang^®^ leuprelin acetate microspheres plus abiraterone (n = 5)							
Gleason grade of 1 (3 + 3)	NA	NA	NA	NA	NA	NA	NA
Gleason grade of 2 (3 + 4)	NA	NA	NA	NA	NA	NA	NA
Gleason grade of 2 (4 + 3)	101.1 (40.0-162.3)	11.8 (0.0, 23.6)	0.5 (0.2, 0.8)	0.1 (0.0, 0.1)	1.8 (0.1, 3.5)	0.0 (0.0, 0.1)	0.092
Gleason grade of 4 (4 + 4, 3 + 5, 5 + 3)	115.5 (12.5-218.4)	2.5 (1.5, 3.5)	0.2 (0.0, 0.4)	0.0 (0.0, 0.0)	0.0 (0.0, 0.0)	0.0 (0.0, 0.0)	0.132
Gleason grade of 5 (4 + 5, 5 + 4, 5 + 5)	NA	NA	NA	NA	NA	NA	NA

The analyses of subgroup of Boennuokang^®^ leuprelin acetate microspheres plus flutamide were not conducted for the number of patients. Data were shown using median (minimum, maximum). PSA, prostate-specific antigen; M1, 1 month after surgery; M3, 3 months after surgery; M6, 6 months after surgery; M12, 12 months after surgery; NA, Not Applicable.

### 3.7 GFR, alkaline phosphatase, and albumin at different time points

GFR did not vary among different time points (*P*=0.441) (**Supplementary Figure 2A**). However, alkaline phosphatase before neoadjuvant therapy, at M1, at M3, at M6, and at M12 was different and showed an increasing trend over time (*P*=0.029); while its level remained within the range of clinical reference (**Supplementary Figure 2B**). No difference was observed in albumin among different time points (*P*=0.548) (**Supplementary Figure 2C**).

### 3.8 Adverse events at different time points

Erectile dysfunction and loss of libido was the most common adverse event, with incidences of 84.9% during neoadjuvant therapy, 79.2% at M1, 71.7% at M3, 67.9% at M6, and 56.6% at M12. Other adverse events, including hot flash, fatigue, osteoporosis, and gastrointestinal reactions, are listed in **Table 6**.

**Table 6:** Adverse events.

Items	Patients (N = 53)
Adverse events during neoadjuvant therapy	
Erectile dysfunction and loss of libido	45 (84.9)
Hot flash	2 (3.8)
Fatigue	2 (3.8)
Osteoporosis	1 (1.9)
Gastrointestinal adverse events	1 (1.9)
Adverse events at M1	
Erectile dysfunction and loss of libido	42 (79.2)
Hot flash	1 (1.9)
Fatigue	1 (1.9)
Adverse events at M3	
Erectile dysfunction and loss of libido	38 (71.7)
Hot flash	6 (11.3)
Adverse events at M6	
Erectile dysfunction and loss of libido	36 (67.9)
Hot flash	1 (1.9)
Adverse events at M12	
Erectile dysfunction and loss of libido	30 (56.6)

Data were shown using No. (%). M1, 1 month after surgery; M3, 3 months after surgery; M6, 6 months after surgery; M12, 12 months after surgery.

## 4 Discussion

Leuprorelin, an LHRH agonist, is recommended for the treatment of advanced patients with prostate cancer, while some previous studies have suggested that its utilization as neoadjuvant therapy may be promising for patients with high-risk prostate cancer (9, 17–19). It was reported that leuprorelin-based treatment as neoadjuvant therapy reduced PSA level in patients with high-risk prostate cancer (9, 13, 18, 19). Similarly, this study found that the median PSA level was 34.1 ng/mL before neoadjuvant therapy, then decreased to 0.8 ng/mL after neoadjuvant therapy, and further dropped to 0.0 ng/mL at M12 in patients with high-risk prostate cancer who received Boennuokang^®^ leuprorelin acetate microspheres-based treatment as neoadjuvant therapy. The possible reason could be: leuprorelin inhibits pituitary and gonadal function to decrease the production of androgen, and the latter is positively related to PSA (7, 20).

Testosterone is utilized to evaluate castration adequacy for patients with prostate cancer who receive androgen deprivation therapy (21). According to previous studies, leuprorelin-based neoadjuvant treatment reduced the level of testosterone in patients with high-risk prostate cancer (10, 22). Similarly, this study found that testosterone tended to be decreased after neoadjuvant Boennuokang^®^ leuprorelin acetate microspheres-based treatment in patients with high-risk prostate cancer, but did not achieve statistical significance. The possible explanation could be: the sample size of this study was relatively small, which weakened statistical power. In addition, the testosterone level before neoadjuvant therapy was relatively low [median (25^th^, 75^th^): 31.5 (18.8, 366.4) ng/dL], suggesting that patients might have hypogonadism prior to the treatment imitation, which could impact the results. Consequently, further investigation is needed.

The increased prostate volume is linked with the elevated difficulty of surgery and increased risks of complications (23, 24). Previously, it has been reported that leuprorelin-based treatment as neoadjuvant therapy decreased the prostate volume in patients with prostate cancer (19, 25). Similarly, this study revealed that the median (25^th^-75^th^) prostate volume was reduced from 40.5 (33.4-55.2) mL to 30.2 (25.2-40.2) mL after receiving Boennuokang^®^ leuprorelin acetate microspheres-based treatment as neoadjuvant therapy in patients with high-risk prostate cancer. Hence, it was speculated that Boennuokang^®^ leuprorelin acetate microspheres-based treatment as neoadjuvant therapy might reduce the surgical complexity and improve the postoperative outcomes of patients with high-risk prostate cancer.

Positive surgical margin, with an incidence of 48.6%-51.2%, is associated with elevated possibilities of biological recurrence and cancer-related mortality in patients with high-risk prostate cancer receiving radical prostatectomy (6, 26–28). In this study, the incidence of positive surgical margin was 32.1% in patients with high-risk prostate cancer who received Boennuokang^®^ leuprorelin acetate microspheres-based treatment as neoadjuvant therapy, which was lower than that in patients without neoadjuvant therapy in previous studies (48.6%-50.8%) (6, 26, 27). These findings suggested that Boennuokang^®^ leuprorelin acetate microspheres-based treatment as neoadjuvant therapy might reduce the incidence of positive surgical margin in patients with high-risk prostate cancer. Besides, one study showed that neoadjuvant androgen-deprivation therapy did not reduce the positive surgical margin rate in patients with clinical T3 tumors (29), suggesting that patients with higher clinical T stages might not benefit from this treatment. In this study, 34.0% of patients were clinical T3/4 cases and the forward-stepwise multivariate logistic regression analysis showed that higher clinical T stage was independently related to increased likelihood of positive surgical margin in patients with high-risk prostate cancer receiving Boennuokang^®^ leuprorelin acetate microspheres-based treatment as neoadjuvant therapy. These findings suggested that patients with high-risk prostate cancer and clinical T3/4 stages might not derive significant benefit from Boennuokang^®^ leuprorelin acetate microspheres-based treatment as neoadjuvant therapy. However, this hypothesis needed further validation.

In this study, most patients (88.7%) received Boennuokang^®^ leuprorelin acetate microspheres combined with bicalutamide, with fewer patients receiving other combination therapies. The possible reason could be that bicalutamide was included in medical insurance coverage and patients were likely to select medications within the insured formulary (30, 31).

Leuprorelin shows a certain possibility of inducing adverse events in the kidney and liver (32, 33). Thus, GFR, alkaline phosphatase, and albumin were detected in this study to reflect renal and liver functions in patients with high-risk prostate cancer who received Boennuokang^®^ leuprorelin acetate microspheres-based treatment as neoadjuvant therapy. The results exhibited that GFR and albumin were not different before neoadjuvant therapy and after surgery in these patients. In addition, alkaline phosphatase was elevated over time, but its level remained within the range of clinical reference, and thus its elevation was clinically insignificant. These findings suggested that the adverse impact of Boennuokang^®^ leuprorelin acetate microspheres-based treatment as neoadjuvant therapy on kidney and liver was minimal in patients with high-risk prostate cancer.

Given the anti-androgen effect of leuprorelin, the most common adverse effect of leuprorelin was erectile dysfunction and loss of libido in patients with prostate cancer, with the incidence ranging from 13.6% to 90.9% (9, 34–36). In this study, erectile dysfunction and loss of libido was the most common adverse event and its incidence was 84.9% during neoadjuvant therapy in patients with high-risk prostate cancer who received Boennuokang^®^ leuprorelin acetate microspheres-based neoadjuvant therapy, which was in the range of previous studies (9, 35, 36). Besides, incidences of other adverse events were low during neoadjuvant therapy in this study, such as hot flash (3.8%), fatigue (3.8%), osteoporosis (1.9%), and gastrointestinal reactions (1.9%), which were continuously reduced after surgery. Hence, the adverse effect of Boennuokang^®^ leuprorelin acetate microspheres-based treatment as neoadjuvant therapy was reversible, and its overall safety was acceptable in patients with high-risk prostate cancer.

Neoadjuvant hormonal therapy, including leuprorelin, could induce morphologic changes of prostate cancer tissues, such as atrophy of glands, basal cell prominence, and squamous and transitional cell metaplasia (37, 38). These changes are a considerable challenge in the post-treatment pathological assessment (39). As a result, histological assessment and Gleason score evaluation after Boennuokang^®^ leuprorelin acetate microspheres-based neoadjuvant therapy became more complex, which required further exploration (40).

Limitations still existed in the current study. First, the sample size was relatively small in this study, which weakened statistical power. Therefore, further studies with a larger sample size are required for validation. Second, this was a single-arm study without an untreated control group or other treatment arms. Therefore, the efficacy of Boennuokang^®^ leuprorelin acetate microspheres-based treatment as neoadjuvant therapy compared to non-neoadjuvant treatments or other neoadjuvant regimens remains uncertain in patients with high-risk prostate cancer, which needs future exploration. Third, most (88.7%) patients in this study received Boennuokang^®^ leuprorelin acetate microspheres plus bicalutamide and the number of patients receiving Boennuokang^®^ leuprorelin acetate microspheres plus other agents was small. Thereby, subgroup analysis of patients who received different neoadjuvant regimens could not show reliable results and the optimal Boennuokang^®^ leuprorelin acetate microspheres-based regimen requires further investigation. Fourth, adhesions are common surgical complications (41). Due to the lack of data on surgical complications, this study was unable to assess the impact of Boennuokang^®^ leuprorelin acetate microspheres-based treatment as neoadjuvant therapy on surgical complications and future investigations were warranted for this issue. Fifth, the concomitant use of 1^st^ and 2^nd^ generation androgen receptor pathway inhibitors could impact the internal validity of the results. Thus, the findings should be interpreted with caution and future studies are required for verification. Sixth, pre-existing erectile dysfunction or loss of libido prior to neoadjuvant therapy was unclear due to the lack of data in this study, which might influence the evaluation of these issues. Therefore, further validation with more comprehensive data is needed in the future.

In conclusion, Boennuokang^®^ leuprorelin acetate microspheres-based treatment as neoadjuvant therapy achieves reduced PSA, testosterone, and prostate volume, and low positive surgical margin rate in patients with high-risk prostate cancer. In addition, this neoadjuvant regimen may induce sexual dysfunction, but this adverse effect tends to be alleviated over time. Therefore, the safety profiles of this treatment should be validated in these patients. These findings support that the clinical application of Boennuokang^®^ leuprorelin acetate microspheres-based neoadjuvant treatment may be promising for improving preoperative tumor shrinkage, reducing surgical complexity, and enhancing postoperative outcomes in patients with high-risk prostate cancer. With appropriate management of potential adverse effects, the long-term benefits of this neoadjuvant treatment may outweigh the associated risks, making it a promising therapeutic option for these patients. Future studies should focus on assessing longitudinal changes in tumor microenvironment, biomarkers of prognosis, androgen receptor expression, tumor driver expression, and immune infiltrate levels before and after treatment. Additionally, longitudinal evaluations of blood and systemic immune profiles as well as cytokine levels will also help provide a more comprehensive understanding of treatment mechanisms and prognosis in patients with high-risk prostate cancer undergoing this neoadjuvant therapy.

## Data Availability

The original contributions presented in the study are included in the article/**Supplementary Material**. Further inquiries can be directed to the corresponding author.
