# Supplementary material for: Real-world analysis of leuprorelin acetate microspheres-based neoadjuvant therapy for patients with high-risk prostate cancer
Source: Front Oncol. 2025 Mar 19;15:1520370. doi: 10.3389/fonc.2025.1520370 (PMC11961931; doi:10.3389/fonc.2025.1520370)
Supplement: Supplementary file 1 [file DataSheet1.docx]

***Supplementary Materials***

**Supplementary Table 1.** Baseline Gleason grade in patients with biochemical recurrence.

| Baseline Gleason grade | Patients with biochemical recurrence | | |
| --- | --- | --- | --- |
|  | M3 (n = 5) | M6 (n = 3) | M12 (n = 4) |
| 1 (3+3) | 0 (0.0) | 0 (0.0) | 0 (0.0) |
| 2 (3+4) | 0 (0.0) | 0 (0.0) | 0 (0.0) |
| 2 (4+3) | 1 (20.0) | 0 (0.0) | 1 (25.0) |
| 4 (4+4, 3+5, 5+3) | 1 (20.0) | 1 (33.3) | 0 (0.0) |
| 5 (4+5, 5+4, 5+5) | 3 (60.0) | 2 (66.7) | 3 (75.0) |

Data were shown using No. (%). M3, 3 months after surgery; M6, 6 months after surgery; M12, 12 months after surgery.

**Supplementary Table 2.** Details of 10 patients with downstaging at surgery.

| No. | Age (years) | BMI (kg/m^2^) | Smoke history | Hypertension | Diabetes | Pre-treatment Gleason grade | Pre-treatment T stage | Pre-treatment TNM stage | Post-treatment T stage | Post-treatment TNM stage |
| --- | --- | --- | --- | --- | --- | --- | --- | --- | --- | --- |
| 1 | 69 | 22.0 | No | Yes | Yes | 4+5=9 | T3b | T3bN0M0 | T3a | T3aN0M0 |
| 2 | 71 | 23.4 | No | No | No | 4+5=9 | T2c | T2cNxM1b | T2b | T2bNxM1b |
| 3 | 67 | 17.3 | Yes | No | No | 5+5=10 | T3b | T3bNxMo | T3a | T3aNxM0 |
| 4 | 75 | 21.8 | No | No | No | 4+5=9 | T3a | T3aNxMx | T2c | T2cNxMx |
| 5 | 71 | 23.2 | No | No | No | 4+4=8 | T3b | T3bN1M1b | T3a | T3aN1M1b |
| 6 | 72 | 26.9 | No | No | No | 3+4=7 | T3b | T3bN0M0 | T3a | T3aN0M0 |
| 7 | 75 | 26.3 | No | No | No | 5+4=9 | T4 | T4N1M0 | T3b | T3bN1M0 |
| 8 | 79 | 22.7 | No | No | No | 4+3=7 | T3b | T3bN0M0 | T2 | T2N0M0 |
| 9 | 68 | 25.5 | No | No | No | 5+4=9 | T3b | T3bN1Mx | T3a | T3aN1Mx |
| 10 | 77 | 30.4 | No | Yes | No | 5+4=9 | T3b | T3bNxMx | T3a | T3aNxMx |

BMI, body mass index; T, tumor, N, node; M, metastasis; TNM, tumor-node-metastasis.

**Supplementary Table 3.** Association of neoadjuvant therapy regimen with Gleason grade.

| Gleason grade | Boennuokang^®^ leuprelin acetate microspheres plus bicalutamide | Boennuokang^®^ leuprelin acetate microspheres plus abiraterone | | Boennuokang^®^ leuprelin acetate microspheres plus flutamide | *P* value |
| --- | --- | --- | --- | --- | --- |
| 1 (3+3), n (%) | 2 (4.3) | | 0 (0.0) | 0 (0.0) | 0.715 |
| 2 (3+4), n (%) | 3 (6.4) | | 0 (0.0) | 0 (0.0) |  |
| 2 (4+3), n (%) | 14 (29.8) | | 2 (40.0) | 0 (0.0) |  |
| 4 (4+4, 3+5, 5+3), n (%) | 7 (14.9) | | 2 (40.0) | 0 (0.0) |  |
| 5 (4+5, 5+4, 5+5), n (%) | 21 (44.7) | | 1 (20.0) | 1 (100.0) |  |

Data were shown using No. (%).

**
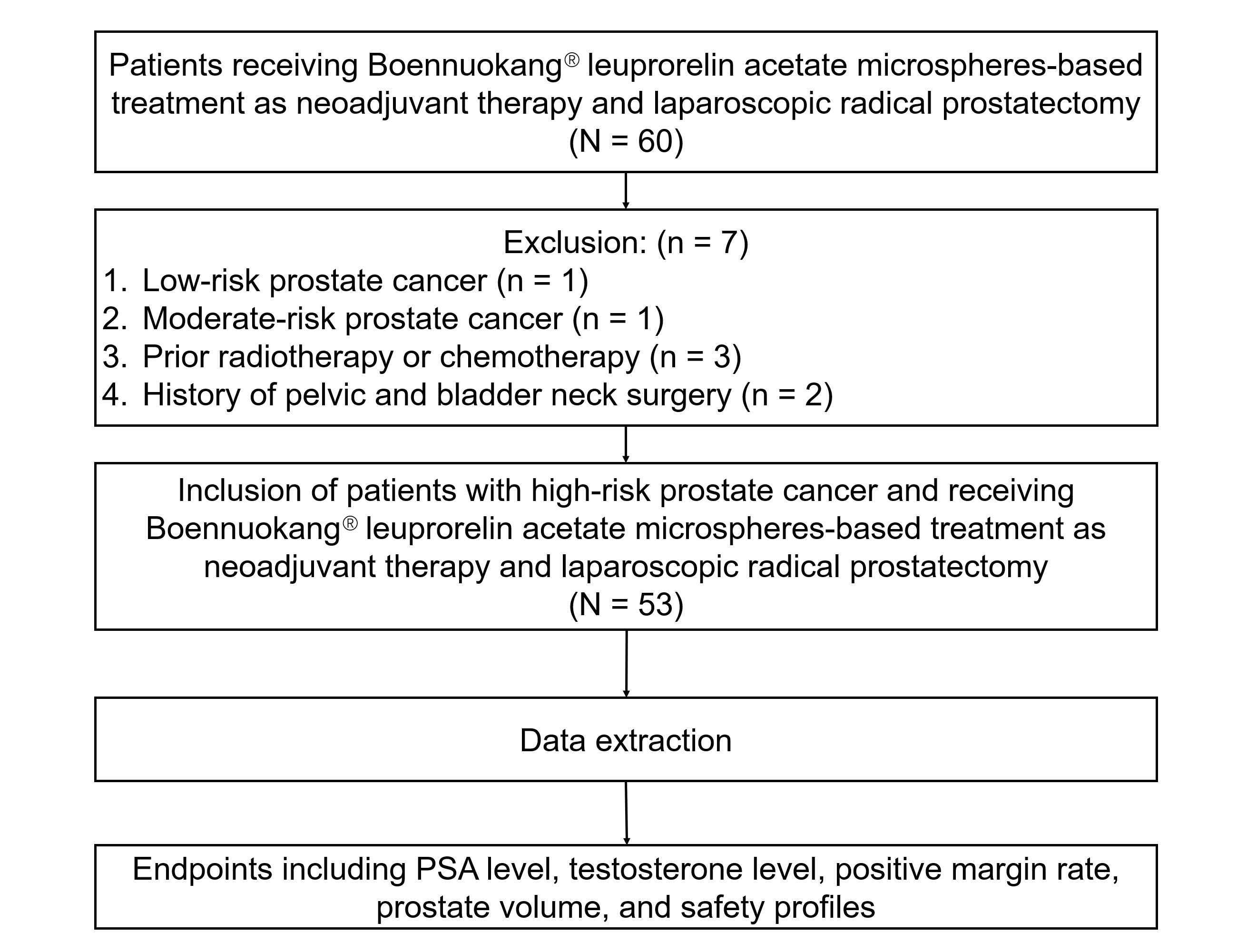
**

**Supplementary Figure 1.** A schema of study design.


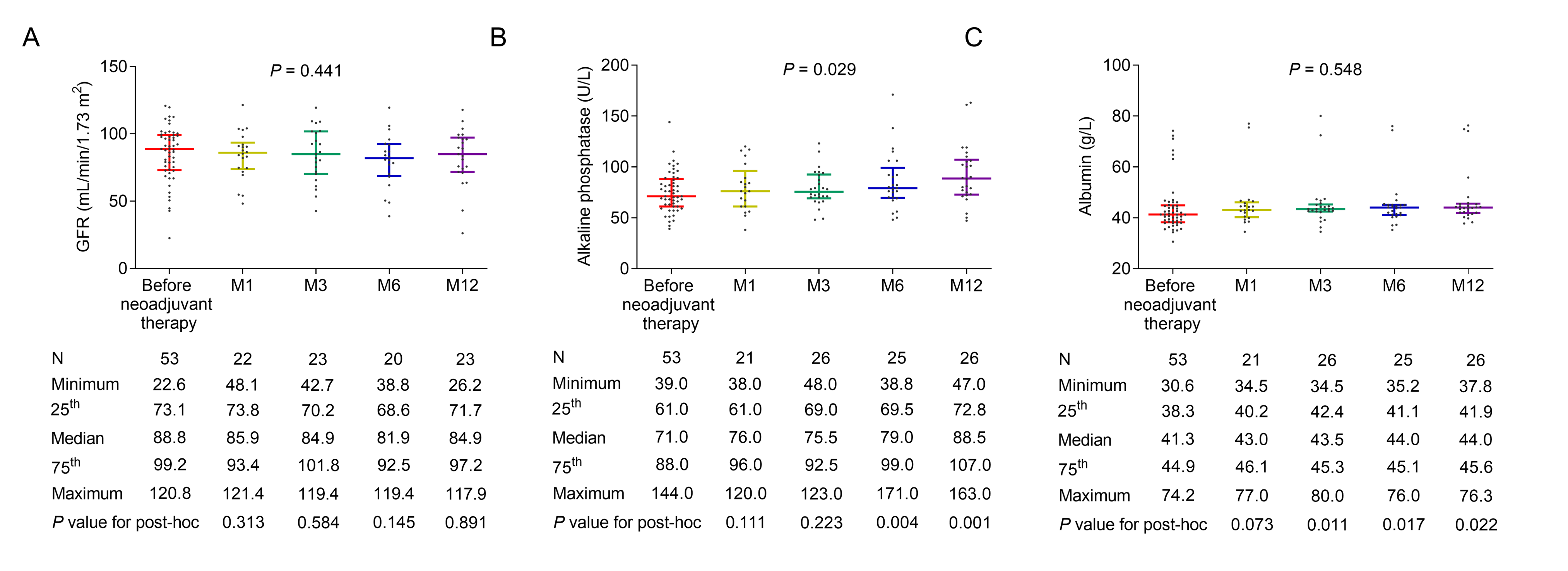


**Supplementary Figure 2.** Levels and trends of GFR, alkaline phosphatase, and albumin over time in patients with high-risk prostate cancer who received Boennuokang^®^ leuprorelin acetate microspheres-based treatment as neoadjuvant therapy. GFR (**A**), alkaline phosphatase levels (**B**), and albumin levels (**C**) before neoadjuvant therapy, at M1, at M3, at M6, and at M12.
